# Successful Bridge-to-Recovery Treatment in a Young Patient with Fulminant Eosinophilic Myocarditis: Roles of a Percutaneous Ventricular Assist Device and Endomyocardial Biopsy

**DOI:** 10.1155/2019/8236735

**Published:** 2019-07-02

**Authors:** Saki Hasegawa-Tamba, Keiki Sugi, Yodo Gatate, Kanako Sugiyama, Toshihiro Muramatsu, Shigeyuki Nishimura, Masanori Yasuda, Kenji Fukushima, Shintaro Nakano

**Affiliations:** ^1^Department of Cardiology, International Medical Center, Saitama Medical University, Saitama, Japan; ^2^Division of Pathology, Saitama Medical University, Saitama, Japan; ^3^Department of Nuclear Medicine, International Medical Center, Saitama Medical University, Saitama, Japan

## Abstract

Eosinophilic myocarditis (EM) is a rare condition characterized by myocardial eosinophilic infiltration due to various underlying etiologies. The patient with EM may benefit from appropriate use of mechanical circulatory support (MCS) that acts as a bridge to myocardial recovery in response to effective immunosuppressive therapy. A 16-year-old boy presented with cardiogenic shock due to fulminant myocarditis, for which a percutaneous ventricular assist device (PVAD) was immediately inserted. Based on the histological diagnosis of EM, immunosuppressive therapy was immediately commenced, leading to improvement of left-ventricular ejection fraction (27% to 47%). The PVAD was successfully removed on day 7. Cardiac magnetic resonance imaging and dual-tracer myocardial scintigraphy suggested limited extent of irreversible myocardial damage. For fulminant EM, the short-term use of PVAD, together with immunosuppressive therapy guided by an immediate histological investigation, may be an effective bridging strategy to myocardial recovery.

## 1. Introduction

Eosinophilic myocarditis (EM) is a rare condition characterized by myocardial eosinophilic infiltration due to various underlying etiologies [1–4]. Although EM manifests as fulminant myocarditis that is often fatal [5, 6], the patient may benefit from appropriate use of mechanical circulatory support (MCS) that acts as a bridge to myocardial recovery in response to effective immunosuppressive therapy. Veno-arterial extracorporeal membrane oxygenation (VA-ECMO) is an established therapeutic MCS option for patients in severe cardiogenic shock due to fulminant myocarditis because it facilitates rapid induction of stable hemodynamics via a femoral approach [7–10]. Despite VA-ECMO, however, the mortality rate associated with cardiogenic shock is still high [11], presumably because of its unfavorable effects on hemodynamics such as an increase in the afterload. The Impella 2.5 system (Abiomed, Danvers, MA, USA), a miniature percutaneous left-ventricular (LV) assist device (PVAD), was launched in our county in September 2017. It equips axial-flow pumps from the left ventricle to the ascending aorta to unload the left ventricle as well as provide flow support up to 2.5 L/min [8, 12].

We present the case of a young boy who survived fulminant EM with successful short-term MCS using PVAD as a bridge to myocardial recovery in response to histology-guided immunosuppressive therapy.

## 2. Case Presentation

**Figure 1:**
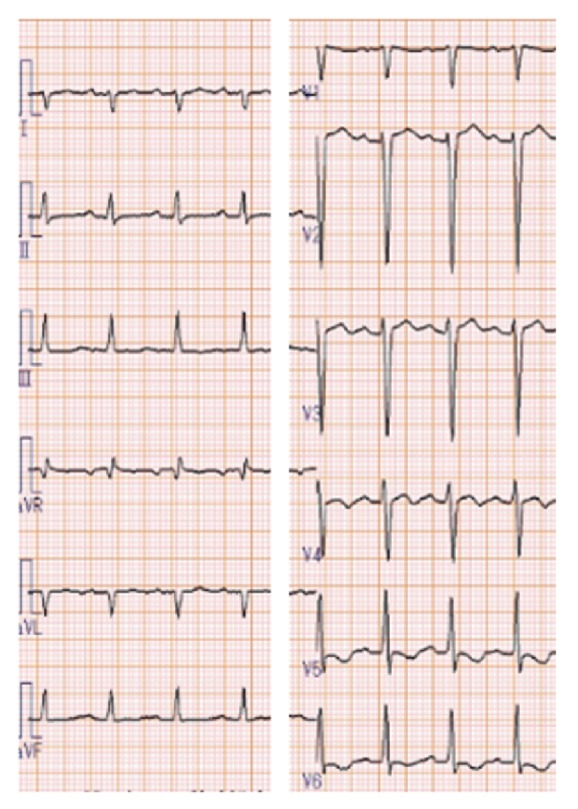
*Electrocardiogram on admission*. Electrocardiogram shows tachycardia, sinus rhythm, right axis deviation, nonspecific ST-T change, and clockwise rotation.

**Figure 2:**
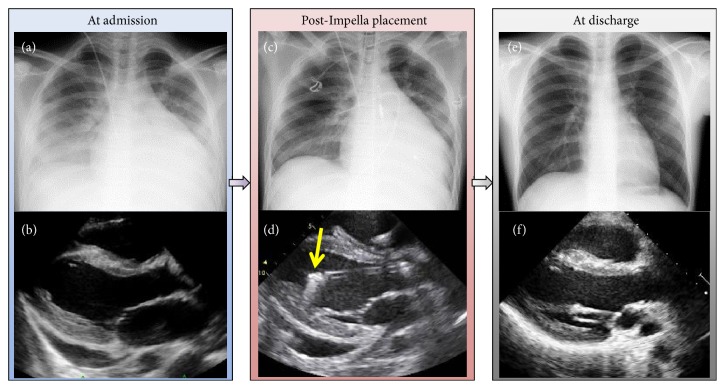
*Findings on admission and after Impella placement*. (a) Pre-Impella placement chest radiograph shows prominent pulmonary edema with an enlarged cardiac shadow. (b) At admission (pre-Impella placement), echocardiography, parasternal long-axis view, shows an extensively thickened left ventricle with enhanced echogenicity and a modest amount of pericardial effusion [left-ventricular ejection fraction (LVEF) 42%]. (c) Post-Impella placement chest radiography shows less pulmonary edema. (d) Post-Impella placement echocardiography, parasternal long-axis view, shows Impella inlet parts with an acoustic shadow in the proper position (yellow arrow) (LVEF 27%). (e) At discharge, chest radiography shows an almost normal cardiac shadow. (f) At discharge, echocardiography, parasternal long-axis view, shows improved contractility without pericardial effusion (LVEF 60%).

A previously healthy, athletic 16-year-old boy (body surface area 1.82 mm^2^) was transferred to our cardiac institution with a 9-day history of a sustained high fever complicated by diarrhea and abdominal pain. He had no history of allergic disease such as bronchial asthma, or known drug allergies. He had received oral cefdinir for a few days for suspected bacterial enterocolitis two weeks previously. His symptoms had been resistant to the antibiotics and antipyretic medications given in the prior tertiary center. Upon arrival at our center, he exhibited cardiogenic shock with a body temperature of 40.5°, heart rate 107 beats/min (irregular), systolic pressure 90, and diastolic pressure ranging from 30 to 75 mmHg (measured by oscillometric method, unstable), and respiratory rate 48/min. He was mostly conscious but occasionally stuporous. No jugular distension, limb edema, or skin rash was observed. His peripheral body was cold despite his body trunk being warm. On auscultation, he had regularly irregular muffled heart sounds without a significant murmur, and some rales bibasally. The electrocardiogram revealed tachycardia, sinus rhythm, right axis deviation, nonspecific ST-T change, and clockwise rotation (Figure 1). His chest radiograph showed prominent pulmonary edema, accumulation of pleural effusion, and an enlarged cardiac shadow (Figure 2(a)). Echocardiography showed LV systolic dysfunction—LV end-diastolic volume (LVEDV) and end-systolic volume (LVESV) = 130/76 mL; LV ejection fraction (LVEF) 42%—a mildly thickened and echogenic LV wall, and a modest amount of pericardial effusion, without visible intraventricular thrombus (Figure 2(b)). Blood tests showed respiratory alkalosis, high concentration of markers of cardiac injury (troponin I 1843.6 pg/mL [upper limit of normal: 26.2 pg/mL]), heart failure (plasma brain natriuretic peptide 2671.1 pg/mL [18.4 pg/mL]), evidence of an inflammatory response (white blood cell count 8.5 × 10^3^/*μ*L with neutrophils 93% and eosinophils 1.5% and serum C-reactive protein 29 mg/dL), mild hepatic failure (aspartate aminotransferase 145 U/L [38 U/L] and alanine aminotransferase 100 U/L [44 U/L]), and almost unimpaired renal function (blood urea nitrogen 26 mg/dL [20 mg/dL] and creatinine 1.2 mg/dL [1.08 mg/dL]). The myeloperoxidase-anti-neutrophil cytoplasmic antibody was below the detectable range.

Despite inotropic support and fluid resuscitation, his systolic blood pressure suddenly fell to 80 mmHg in the middle of the initial evaluation. Based on the clinical diagnosis of cardiogenic shock, an Impella 2.5 was immediately inserted into the right femoral artery via a 12-Fr sheath, followed by a right-ventricular (RV) endomyocardial biopsy (EMB) via the right internal jugular. Intact coronary arteries were subsequently confirmed by coronary angiography. Chest radiography after Impella placement showed alleviated pulmonary edema (Figure 2(c)) together with elevation of the mean arterial pressure from 51 to 72 mmHg, although the LVEF was decreased to 27% (Figure 2(d); Supplementary Movie 1). Because of his high sustained fever, fluid resuscitation with extracellular fluid and blood products (approximately 6 L/day) was required to maintain the mean arterial pressure >55 mmHg. However, the mean pulmonary wedge pressure was mildly elevated (10–13 mmHg) without the patient displaying radiographic pulmonary edema.

**Figure 3:**
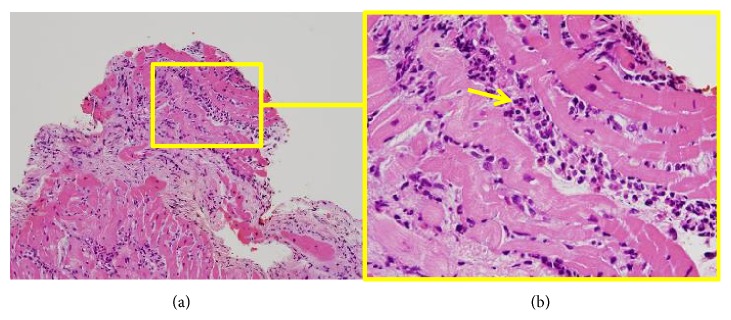
*Histological findings of right-ventricular endomyocardial biopsy specimen on admission*. Hematoxylin-eosin staining (formalin-fixed; paraffin-embedded) shows damaged myocardium and infiltration of degranulated eosinophils (arrow, right), lymphocytes, and neutrophils. (a) ×20. (b) ×60.

Within 48 h after admission, eosinophilic myocarditis was diagnosed based on the histological findings (Figure 3). Immunosuppressive therapy consisting of methylprednisolone 1000 mg/day for 3 days followed by prednisolone 1 mg/kg/day and azathioprine 2 mg/kg/day was commenced, which improved his LV systolic function in the short term (LVEF 47% by day 6) and decreased the body temperature, leading to hemodynamic stabilization. The doses of inotropes required to maintain stable hemodynamics during Impella use were decreased by day 4 (maximum dose of dobutamine was 2.2 *μ*g/kg/min and of dopamine 1.5 *μ*g/kg/min on days 1 and 2).

**Figure 4:**
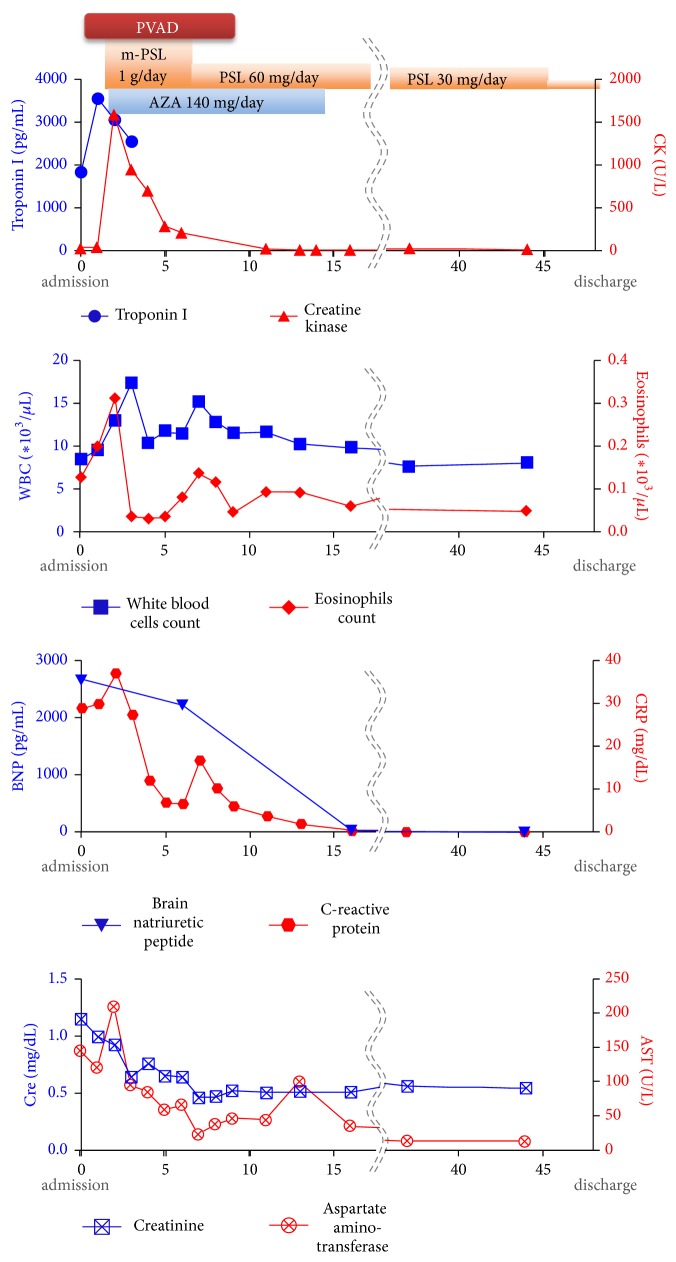
*Changes in blood markers during hospitalization*. Graphs showing changes in serum troponin I and creatine-kinase concentrations, white blood cell and eosinophil counts, plasma brain natriuretic peptide, serum C-reactive protein, serum creatinine, and serum aspartate amino transferase concentrations, together timing of with mechanical circulatory support and immunosuppressive therapy. PVAD, percutaneous ventricular assist device; m-PSL, methyl-prednisolone; PSL, prednisolone.

He was gradually, day by day, weaned from the Impella, decreasing from the P8 to P2 level without showing marked end-organ failure. The Impella was surgically removed on day 7, with his postremoval blood pressure at 120/70 mmHg and heart rate at 65 beats/min on with minimal inotropic support (dobutamine and dopamine, 3.0 *μ*g/kg/min each) to avoid sudden hemodynamic deterioration immediately after removal of the MCS. He was extubated on day 8, followed by initiation of antiheart failure medications (e.g., angiotensin-converting-enzyme inhibitor and *β*-blocking agent) to prevent further remodeling. The follow-up EMB, performed 2 weeks after his admission, revealed improved infiltration of eosinophils and lymphocytes and some degree of fibrosis (Supplementary Figure 1). Azathioprine was tapered off, whereas prednisolone dose was gradually tapered to 30 mg/day. He was discharged from hospital 6 weeks after the admission without a major complication (Figure 4).

**Figure 5:**
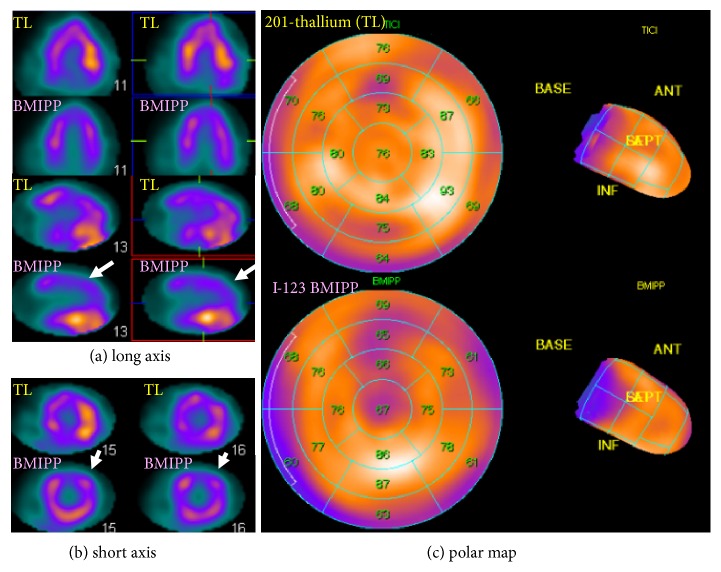
*Thallium-201 and iodine-123 β-methyl-p-iodophenylpentadecanoic acid dual-tracer myocardial scintigraphy 3 weeks after admission*. Slight patchy deficit was found for myocardial thallium uptake in the anterolateral wall and further decreased uptake of BMIPP, which was consistent with a mild perfusion-metabolism mismatch (white arrows). (a) Long-axis 2D view. (b) Short axis 2D view. (c) Polar map. 2D, two-dimensional; ANT, anterior; INF, inferior; TL, thallium.

Thallium-201 and iodine (I)-123 *β*-methyl-p-iodophenylpentadecanoic acid (BMIPP) dual-tracer myocardial scintigraphy 3 weeks after admission showed a patchy deficit of perfusion at the anterolateral wall and a discordantly larger deficit of fatty acid metabolism (perfusion-metabolism mismatch), suggesting regional myocardial damage with potential reversibility (Figure 5).

**Figure 6:**
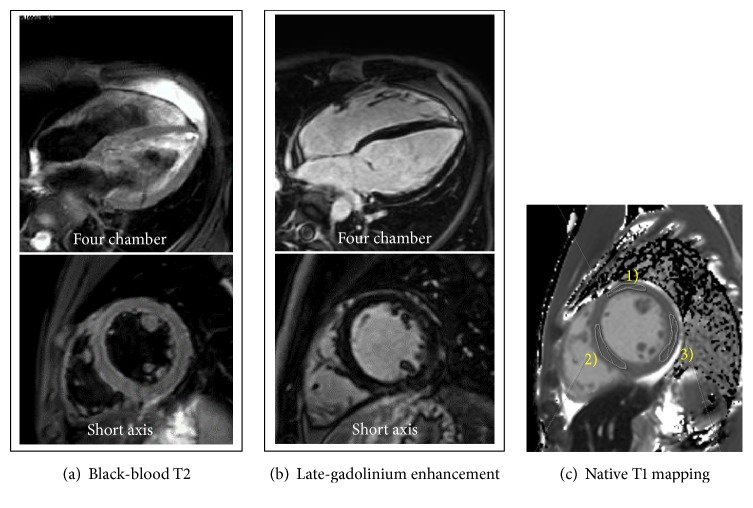
*Cardiac 1.5-T magnetic resonance imaging 4 weeks after admission*. (a) T2-weighted black-blood images show no significant high-intensity signal. (b) Late-gadolinium enhancement shows no significant focal high-intensity signal. (c) Native T1 mapping analysis shows mildly elevated values: (1) anterior wall, 1193.01±36.34 msec, (2) septal wall, 1193.05±32.03 msec, and (3) posterolateral wall, 1198.87±42.86 msec.

Cardiac 1.5-T magnetic resonance imaging (CMR) 4 weeks after admission showed no high-intensity areas on T2-weighted imaging or late-gadolinium enhancement (LGE), but prolonged native T1 mapping at papillary muscle level, indicating myocardial edema (Figure 6; Supplementary Movie 2).

At discharge from the hospital, follow-up chest radiography revealed diminishing cardiomegaly with no pulmonary congestion (Figure 2(e)). At the same time echocardiography showed recovery of contractility with LVEF 60% (Figure 2(f)). The patient was weaned off steroid therapy 5 months after the onset of his myocarditis and showed no evidence of recurrence in the subsequent 7 months.

## 3. Discussion

At the time of presentation to our hospital, we recognized that our patient had complicated acute myocarditis (LVEF <50%), which is associated with a worse prognosis and therefore important to identify on presentation [13]. The patient fully recovered from fulminant EM owing to the bridging MCS treatment with PVAD, combined with immunosuppressive therapy based on the histological findings of immediate EMB. Although his cardiac function was temporarily severely impaired, the patient showed no major end-organ dysfunction, severe pulmonary edema, or irreversible myocardial damage. These favorable outcomes may be attributed to the immediate use of PVAD, which supported antegrade flow and unloaded the LV preload.

### 3.1. Diagnosis and Medical Treatment of Eosinophilic Myocarditis

The proposed underlying etiologies of EM include hypersensitivity reactions [6, 14], autoimmune-mediated disease [e.g., eosinophilic granulomatosis with polyangiitis [15, 16]; hypereosinophilic syndrome or its variants [17–19]], parasitic infections [20], and cancer [19]. However, the cause in a substantial proportion of patients remains unknown, as in our case. Our patient had no history of allergic disease, including bronchial asthma or drug allergy, and the fact that he was negative for myeloperoxidase-anti-neutrophil cytoplasmic antibody makes eosinophilic granulomatosis with polyangiitis unlikely. He had received oral cefdinir; however, it had been ceased a week prior to development of cardiac symptoms. Preceding enterocolitis (viral infection) or oral antibiotics are potential underlying causes for the development of EM; however, the precise causality cannot be determined. In fulminant myocarditis, acute (usually within 2 weeks of symptom onset) worsening of the patient's hemodynamics leads to cardiogenic shock [21–23]. Thus, the clinical course of fulminant EM is often fatal, with in-hospital mortality at approximately one-third or one-half of these patients [5, 6, 24].

Peripheral eosinophilia may be absent in 25% of patients [5], as was the case with our patient. Eosinophilic cationic protein [25] and total immunoglobulin E may play roles in the pathogenesis of EM; however, we did not measure these markers. Only EMB allows a definitive diagnosis of EM [26–28]. The European Society of Cardiology Working Group proposed using EMB as a tool for monitoring and guiding therapy in patients with specific forms of myocarditis, such as EM, that potentially could be treated with immunosuppression [29]. Although no immunosuppressive therapy protocol for fulminant EM has yet been established, previous reports have suggested that steroids are effective (i.e., methylprednisolone 1000 mg/day for 3 days followed by prednisolone (1 mg/kg/day) [30, 31] or azathioprine (2 mg/kg/day) in combination with steroids is effective [32]). These reports on immunosuppressive therapy are consistent with our case in which the patient exhibited an immediate favorable clinical and histological response to the immunosuppressive therapy. Patients with EM may show a nontypical, undefined LGE pattern [5]. We did not perform CMR imaging in the acute phase because the patient needed to rest. A CMR performed 4 weeks later revealed no LGE; we speculate that early initiation of immunosuppressive therapy prevented prominent irreversible fibrotic change.

### 3.2. Mechanical Circulatory Support for Cardiogenic Shock Including Fulminant Myocarditis

The use of VA-ECMO is an attractive MCS option for patients in severe cardiogenic shock [7–10]. However, disadvantageous effects associated with the lack of LV unloading (e.g., increased LV filling, elevated pulmonary capillary pressures, or reduced subendocardial myocardial coronary flow) are of great concern [8, 33].

The Impella 2.5 is a percutaneously inserted device equipped with a miniature, nonpulsatile, axial-flow Archimedes-screw pump that propels it from the left ventricle to the ascending aorta. By unloading the left ventricle and supporting flow, it reduces the total mechanical work, subendocardial ischemia, myocardial oxygen demand, pulmonary capillary wedge pressure, and end-organ malperfusion [8, 34–40]. A durable ventricular assist device is another choice of MCS to unload the left ventricle, but use of this device in patients with fulminant myocarditis is associated with a lower survival rate than percutaneous V-A ECMO, potentially associated with its lack of rapidity to ameliorate hemodynamic instability [41, 42]. Importantly, before insertion of Impella, the presence of intraventricular thrombus should be carefully ruled out to avoid a procedure-related embolism. Such a step is particularly important in patients with EM or those who have experienced apical or extensive myocardial infarction as these patients frequently harbor a mural or apical thrombus. Careful investigation of LV size is also important, as patients with acute myocarditis frequently have a small LV cavity because of myocardial edema; this may lead to suboptimal positioning of the tip of the catheter.

Several previous publications have reported successful results in patients with fulminant myocarditis using the LV Impella, including a 13-year-old boy without a histological diagnosis (Impella 5.0, bridge-to-recovery) [40], a 44-year-old woman with giant cell myocarditis (Impella 2.5, bridge-to-durable LV device) [43], and a 49-year-old woman with suspected autoimmune etiology (Impella CP, bridge-to-recovery) [44]. In our case, continuous fluid resuscitation had been required even after the PVAD insertion as the patient had revealed high sustained fever before immunosuppressive therapy exerted its effect. A combination of “mixed” cardiogenic and circulatory shock can occur in patients with a low cardiac output and an acute inflammatory response; the left-ventricular unloading provided by PVAD was advantageous in refilling intraventricular volume without resulting in development of pulmonary edema.

Our case is specific in that the EMB-driven histological diagnosis played a crucial role in initiating early immunosuppressive therapy, which led to immediately improved myocardial function, allowing us to wean the patient off the Impella. The CMR findings in our case indicated increased extracellular volume (elevated T1 mapping values) but not apparent myocardial fibrosis, as represented by the LGE [45], also suggesting the effectiveness of the bridging MCS strategy in limiting the extent of irreversible myocardial damage.

Combinations of the LV PVAD with other MCS may be effective in various clinical scenarios. For instance, patients with cardiogenic shock accompanied by severely impaired oxygenation may benefit from a combination of LV PVAD and V-A ECMO, by oxygenating and flow-supporting while unloading the left ventricle [46–48]. Another clinical scenario is prolonged cardiogenic shock with RV dysfunction, which is an under-recognized predictor of a poor prognosis [34]. Patients with fulminant/acute myocarditis with severe biventricular failure may benefit from MCS using the LV and RV PVAD [49].

## 4. Conclusion

For fulminant EM, the short-term use of PVAD, together with immunosuppressive therapy guided by an immediate histological investigation, may be an effective bridging strategy to myocardial recovery.

## Supplementary Materials

Supplementary Materials The supplemental materials are movies to demonstrate that the heart function was depressed. Supplementary Figure 1: histological findings of the right-ventricular endomyocardial biopsy specimen 2 weeks after admission.
