# Supplementary material for: Successful Bridge-to-Recovery Treatment in a Young Patient with Fulminant Eosinophilic Myocarditis: Roles of a Percutaneous Ventricular Assist Device and Endomyocardial Biopsy
Source: Case Rep Emerg Med. 2019 Jul 2;2019:8236735. doi: 10.1155/2019/8236735 (PMC6633872; doi:10.1155/2019/8236735)
Supplement: Supplementary Materials — The supplemental materials are movies to demonstrate that the heart function was depressed. Supplementary Figure 1: histological findings of the right-ventricular endomyocardial biopsy specimen 2 weeks after admission. [file 8236735.f1.zip › 8236735.f1/Supplementary figure 1 20190617 final.pptx]

## Slide 1
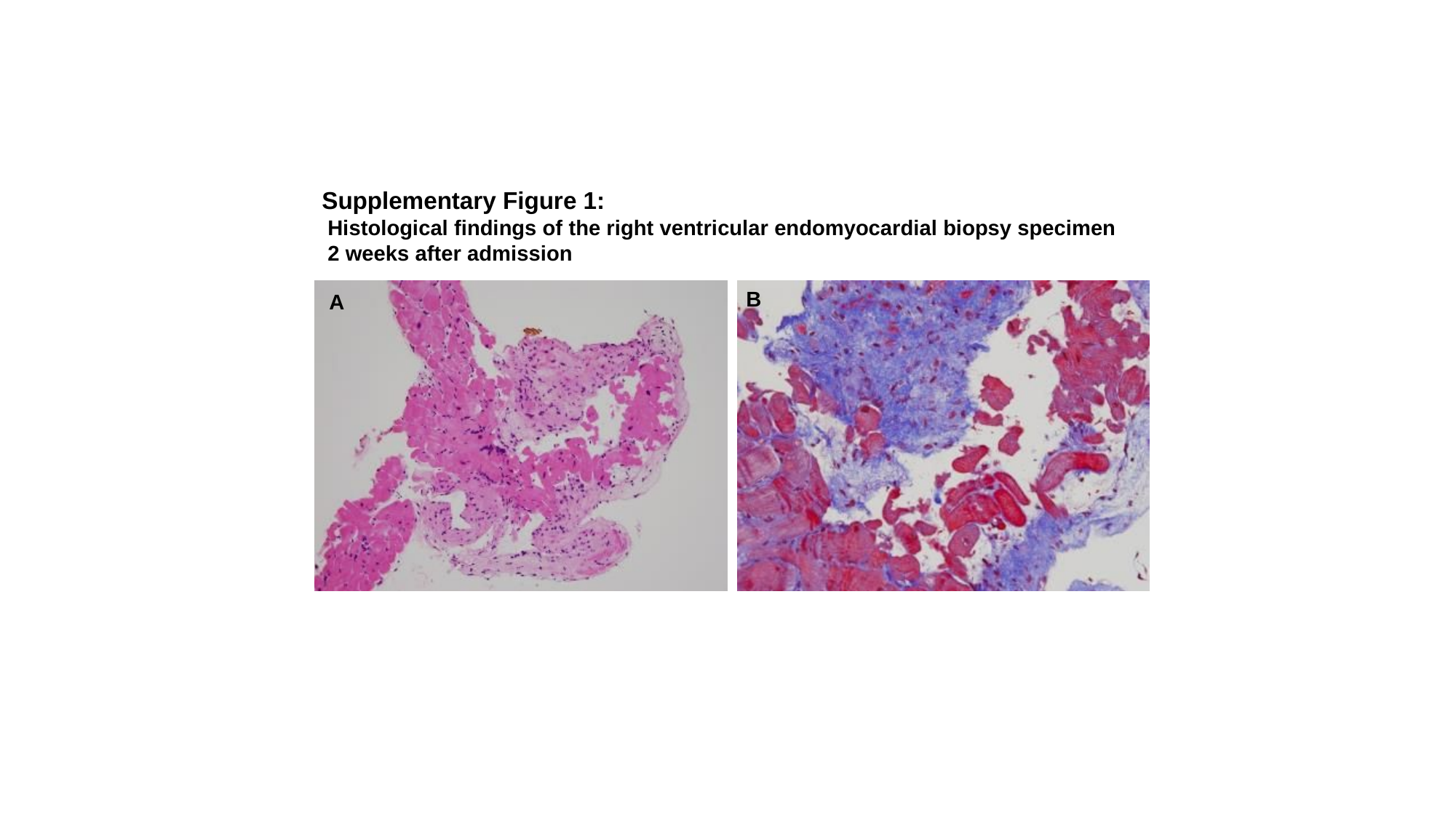

Supplementary Figure 1:
 Histological findings of the right ventricular endomyocardial biopsy specimen
 2 weeks after admission
 B
 A
